# Association between serum ficolin-1 level and disease progression in primary biliary cholangitis

**DOI:** 10.1371/journal.pone.0238300

**Published:** 2020-09-11

**Authors:** Manabu Hayashi, Kazumichi Abe, Masashi Fujita, Atsushi Takahashi, Hideharu Sekine, Hiromasa Ohira

**Affiliations:** 1 Department of Gastroenterology, Fukushima Medical University, Fukushima, Japan; 2 Department of Immunology, Fukushima Medical University, Fukushima, Japan; Emory University School of Medicine, UNITED STATES

## Abstract

Pattern recognition molecules (PRMs) in the complement system contribute to homeostasis as mediators of complement activation. The contribution of PRMs to primary biliary cholangitis (PBC) is unknown. In the current study, we aimed to assess the association between PRMs and the clinical findings of PBC. A total of 122 PBC patients and 20 healthy controls were enrolled. We measured four different PRMs (mannose-binding lectin [MBL], ficolin-1, ficolin-2 and ficolin-3) using stored sera, and retrospectively analyzed the associations between PRMs and laboratory findings, histological findings, and the development of cirrhosis-related conditions. Ficolin-1 levels were significantly higher in the PBC patients than in the healthy controls (152 ng/mL vs 102 ng/mL, P = 0.034), but no significant differences were observed regarding MBL, ficolin-2, and ficolin-3 levels. Ficolin-1 was significantly correlated with alkaline phosphatase (ALP). Low ficolin-1 levels were significantly associated with the development of cirrhosis-related conditions independent for histological stage and ALP levels (hazard ratio: 0.933; 95% confidence interval: 0.875–0.994; P = 0.032). Patients with low levels of ficolin-1 (< 77 ng/mL) had a significantly increased rate of developing cirrhosis-related conditions. Low ficolin-1 levels were associated with disease progression independent of histological stage and ALP levels in patients with PBC.

## Introduction

Primary biliary cholangitis (PBC) is a progressive autoimmune cholestatic liver disease that predominantly affects women [1]. It is characterized by the presence of antimitochondrial antibodies and the progressive destruction of intrahepatic bile ducts. PBC has a mostly slow progression, and the majority of cases are diagnosed at an early stage due to disease awareness; however, disease progression can result in end-stage liver disease and the need for liver transplantation [2]. Ursodeoxycholic acid (UDCA) is the most evaluated drug for PBC; it maintains liver histology [3] and improves long-term survival [4] in PBC patients. However, nearly 40% of patients with PBC are unresponsive to UDCA, and in some cases, the PBC progresses to cirrhosis [5]. Understanding the pathophysiology of PBC is important for deciding on, and administering, the appropriate treatment; however, the pathophysiology of PBC is not yet fully understood.

The complement system plays important roles in innate immunity; maintaining biological homeostasis and acting as immune surveillance [6]. Pattern recognition molecules (PRMs) are an initiator of the complement system activation [7]. PRMs are able to recognize several pathogen-associated molecular patterns, such as immune complexes, carbohydrates, N-glycans, lipopolysaccharides, and sialic acid residues at microbes or endogenous cells. They act as mediators of host defense, and contribute to tissue homeostasis as a scavenger system. Mannose-binding lectin (MBL), ficolin-1 (also known as M-ficolin), ficolin-2 (L-ficolin) and ficolin-3 (H-ficolin) act as PRMs in the lectin pathway. MBL, ficolin-2 and ficolin-3 are produced in the liver, while ficolin-1 is mainly produced in monocytes [8]. Circulating PRMs in the lectin pathway are associated with several disease activities, such as systemic lupus erythematosus and rheumatoid arthritis [9, 10].

Liver injury in PBC is initiated by autoimmune reactions based on genetic and environmental factors [11], and innate immunity is associated with PBC disease progression [12]. Bile acid retention also plays important roles in the pathogenesis of cholestatic liver disease [13]. Recently, the association between complement activation and cholestasis-induced liver injury in mice has been demonstrated [14]. Further, regulation of complement C3 expression by the bile acid receptor FXR has been reported [15]. These findings suggest the possibility of an association between complement activation and PBC. Although MBL levels have been reported to be associated with risk for bacterial infections in cirrhotic patients including those with PBC [16], there have been no reports on the association between PRMs and disease progression of PBC. In the current study, we aimed to assess the associations between PRMs in the lectin pathway and the clinical findings, including disease progression, of PBC.

## Materials and methods

### Patients

This retrospective study enrolled patients who had been diagnosed as having PBC at Fukushima Medical University Hospital (Fukushima, Japan) between September 1988 and June 2017. The diagnosis of PBC was defined by the American Association for the Study of Liver Diseases guidelines [17]. Briefly, PBC was diagnosed when patients met at least two of the following three criteria: (a) chronic elevation of alkaline phosphatase (ALP); (b) presence of anti-mitochondrial M2 antibody or anti-mitochondrial antibodies; and/or (c) compatible or typical histological findings from liver biopsy. Patients were excluded if there was evidence of other liver diseases, such as viral hepatitis, alcoholic liver disease, or non-alcoholic fatty liver disease. Finally, 122 patients with PBC were enrolled. All PBC patients in the current study underwent liver biopsy, which was performed in order to confirm the PBC diagnosis. Serum samples from the PBC patients were obtained at the time of biopsy, and were stored at a temperature of -20°C. We also measured the serum levels of PRM in: patients with chronic hepatitis C without cirrhosis (n = 30); those with hepatitis C-related cirrhosis (n = 13); and those with autoimmune hepatitis (n = 20).

This study was conducted in accordance with the principles outlined in the Declaration of Helsinki. The study protocol, including the use of opt-out consent, was approved by the ethics committee at Fukushima Medical University School of Medicine (date 22 May, 2019; approval number 2019–007). Written informed consent was obtained from all patients for serum and liver biopsy. Written informed consent was also obtained from all control subjects.

### Measurement of pattern recognition molecules

We measured MBL, ficolin-1, ficolin-2 and ficolin-3 in the lectin pathway using an ELISA kit according to the manufacturers’ protocols. All ELISA kits were purchased from Hycult Biotech (Uden, the Netherlands). The diluted serum samples (MBL, 1:50; ficolin-1, 1:20; ficolin-2, 1:10; ficolin-3, 1:150) were used to detect PRMs levels. The measurable concentration ranges in each ELISA kit were as follows: MBL, 0.41–100 ng/ml; ficolin-1, 3.1–200 ng/ml; ficolin-2, 16–1000 ng/ml; and ficolin-3, 8–500 ng/ml.

### Liver histology

We performed percutaneous liver biopsies with an 18-gauge needle. The biopsy specimens were fixed in formalin and embedded in paraffin. Histological findings were evaluated with hematoxylin and eosin staining, and were determined according to the Scheuer staging system [18].

### Development of cirrhosis-related conditions

The development of cirrhosis-related conditions was defined as cirrhosis diagnosed based on biopsy results or at least one of the following events: gastroesophageal varices, ascites, hepatic encephalopathy, hyperbilirubinemia, or hepatocellular carcinoma.

### Statistical analysis

Data are presented as median and interquartile range (IQR), and continuous variables were compared using the Mann–Whitney U test or Kruskal–Wallis test. Multivariate analysis was performed using the Cox proportional hazards model to assess predictors of the development of cirrhosis-related conditions. Cumulative survival without development of cirrhosis-related conditions was calculated using the Kaplan-Meier method, and it was compared using the log-rank test. We considered differences of P < 0.05 to be statistically significant. Analyses of data were performed using the Prism 7.0 software (GraphPad Software, La Jolla, CA, USA) and EZR (Saitama Medical Center, Jichi Medical University, Saitama, Japan), a graphical user interface for R software (The R Foundation for Statistical Computing) [19].

## Results

### Serum levels of pattern recognition molecules in primary biliary cholangitis

The baseline characteristics of the 122 PBC patients at the time of biopsy are shown in Table 1. Twenty patients were cirrhotic at the time of diagnosis (cirrhosis, n = 12; gastrointestinal varices, n = 7; and hyperbilirubinemia, n = 1), and 12 patients developed cirrhosis-related conditions during the follow-up period (gastroesophageal varices, n = 7; cirrhosis diagnosed based on biopsy, n = 1; hepatocellular carcinoma, n = 2; hepatic encephalopathy, n = 1; and hyperbilirubinemia, n = 1). The median follow-up period was 4.1 (interquartile range: 1.0–12.7) years. We also included 20 gender- and age-matched healthy Japanese individuals as controls (median age, 53 years; 17 women [90%]).

**Table 1:** Baseline characteristics in patients with primary biliary cholangitis.

Characteristics	n = 122
Age, years	55 (50–65)
Gender, female (%)	97 (80%)
**Biochemical examination**	
AST (U/L)	46 (31–75)
ALT (U/L)	46 (32–73)
ALP (U/L)	484 (368–665)
GGT (U/L)	144 (72–257)
TB (mg/dL)	0.8 (0.6–1.1)
Alb (g/dL)	4.0 (3.6–4.3)
PT (%)	100 (88–111)
Plt (×10^4^/μL)	20.8 (15.4–24.8)
**Liver histology**	
Scheuer stage (1/2/3/4)	62/29/16/15
**Cirrhosis or cirrhosis-related condition**	
At the time of biopsy	20
During follow-up period	12

Parameters are presented as the median (interquartile range) for continuous variables. Alb, albumin; ALP, alkaline phosphatase; ALT, alanine aminotransferase; AST, aspartate aminotransferase; GGT, γ-glutamyl transpeptidase; Plt, platelet count; PT, prothrombin time; TB, total bilirubin.

**Fig 1:**
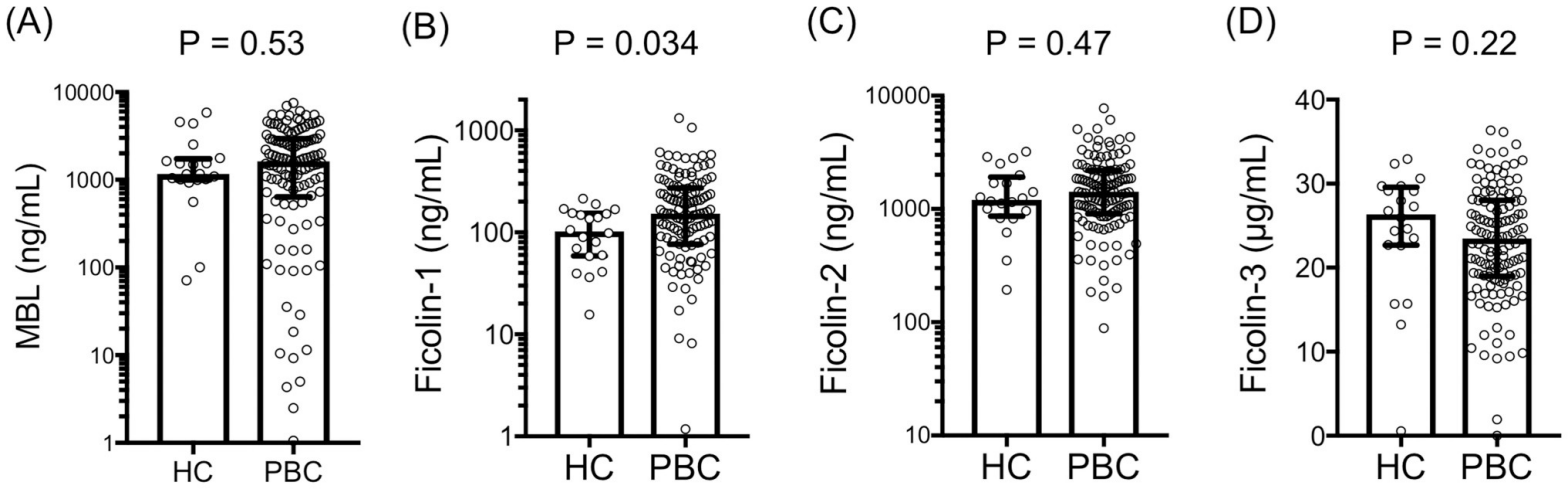
Serum levels of pattern recognition molecules (PRMs) in PBC patients and healthy controls (HC). Serum levels of (A) mannose-binding lectin (MBL), (B) ficolin-1, (C) ficolin-2 and (D) ficolin-3 were measured using ELISA. HC, healthy controls; PBC, primary biliary cholangitis.

The four PRMs in the lectin pathway in both the PBC patients and healthy controls were analyzed (Fig 1). We found that the serum levels of ficolin-1 were significantly higher in the PBC patients than in the controls (152 [77–268] ng/mL vs 102 [59–152] ng/mL; P = 0.034), although no significant differences were observed regarding MBL serum levels (1624 [673–2931] ng/mL vs 1164 [1008–1667] ng/mL; P = 0.53), ficolin-2 (1402 [914–2182] ng/mL vs 1204 [927–1765] ng/mL; P = 0.47) and ficolin-3 (23.4 [19.0–28.0] μg/mL vs 26.3 [22.6–29.2] μg/mL; P = 0.22).

**Fig 2:**
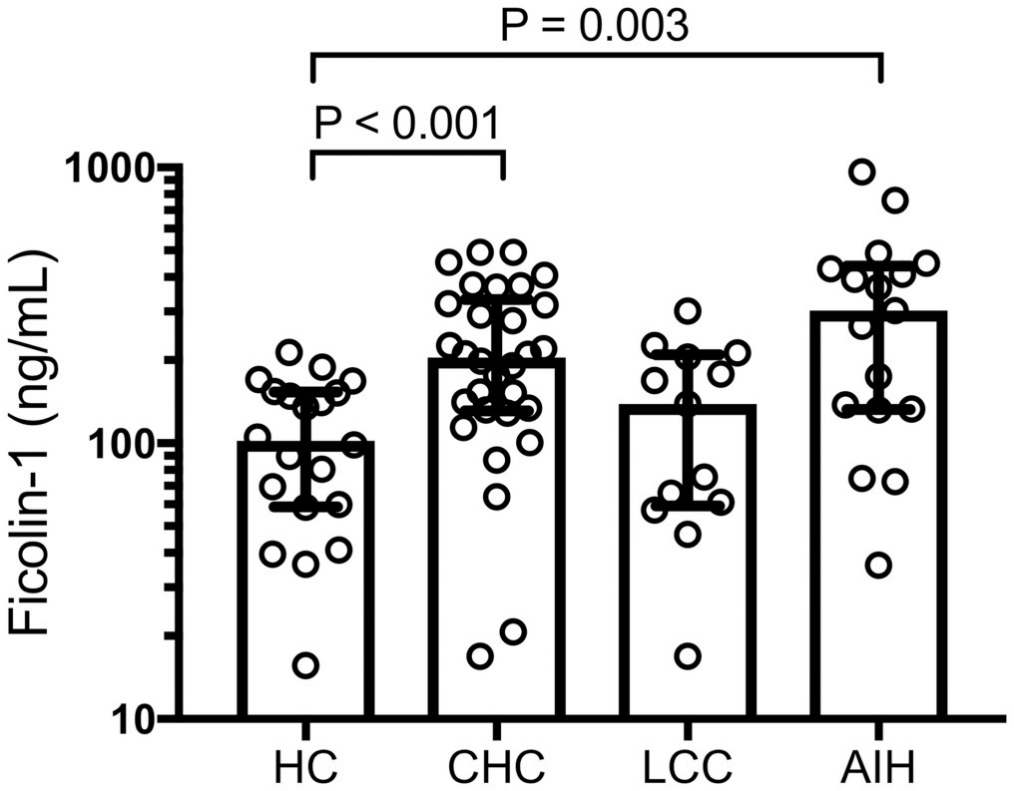
Serum levels of ficolin-1 in patients with hepatitis C infection or autoimmune hepatitis. Ficolin-1 levels in the patients with chronic hepatitis C or autoimmune hepatitis were significantly higher than those of the healthy controls. There was no statistical difference between ficolin-1 levels in the patients with hepatitis C-related cirrhosis and those of the healthy controls. CHC, chronic hepatitis C; HC, healthy control; LCC, liver cirrhosis due to hepatitis C; PBC, primary biliary cholangitis.

Next, we measured ficolin-1 levels in patients with liver diseases other than PBC, such as chronic hepatitis C or autoimmune hepatitis (Fig 2). The ficolin-1 levels in the patients with chronic hepatitis C without cirrhosis (204 [131–332] ng/mL; P < 0.001) or autoimmune hepatitis (302 [132–438] ng/mL; P = 0.003) were significantly higher than those of the healthy controls. The ficolin-1 levels in the patients with hepatitis C-related cirrhosis were higher than those of the healthy controls, although the difference was not statistically significant (139 [59–209] ng/mL; P = 0.41).

### Correlation between pattern recognition molecules and laboratory data

We analyzed the correlations among the four PRMs. Both ficolin-2 and ficolin-3 levels were significantly correlated in the PBC patients (r = 0.49, P < 0.001), although the other PRMs did not show any significant correlations.

**Fig 3:**
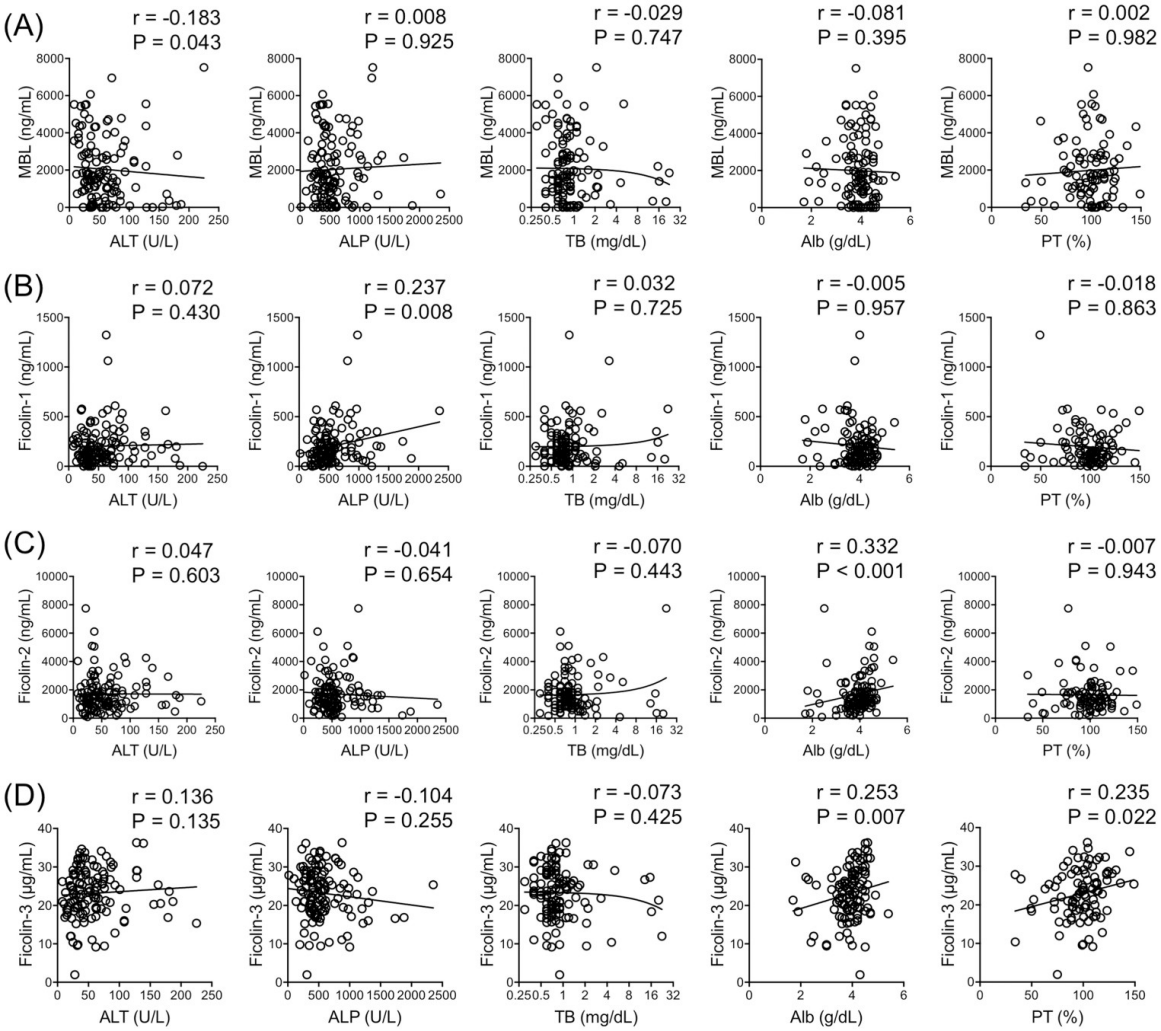
Correlations between laboratory data and (A) mannose-binding lectin (MBL), (B) ficolin-1, (C) ficolin-2 and (D) ficolin-3 levels. Correlations were evaluated using the Spearman Rank correlation coefficient. ALP, alkaline phosphatase; ALT, alanine aminotransferase; MBL, mannose-binding lectin; PT, prothrombin time; TB, total bilirubin.

The correlations of the PRMs with laboratory data were analyzed (Fig 3). MBL levels were significantly correlated with alanine aminotransferase (r = -0.183. P = 0.043); ficolin-1 levels were significantly correlated with ALP (r = 0.237, P = 0.008); ficolin-2 levels were significantly correlated with albumin (r = 0.332, P < 0.001); and ficolin-3 levels were significantly correlated with both albumin (r = 0.253, P = 0.007) and prothrombin time (r = 0.235, P = 0.022).

### Association between pattern recognition molecules and histology

**Fig 4:**
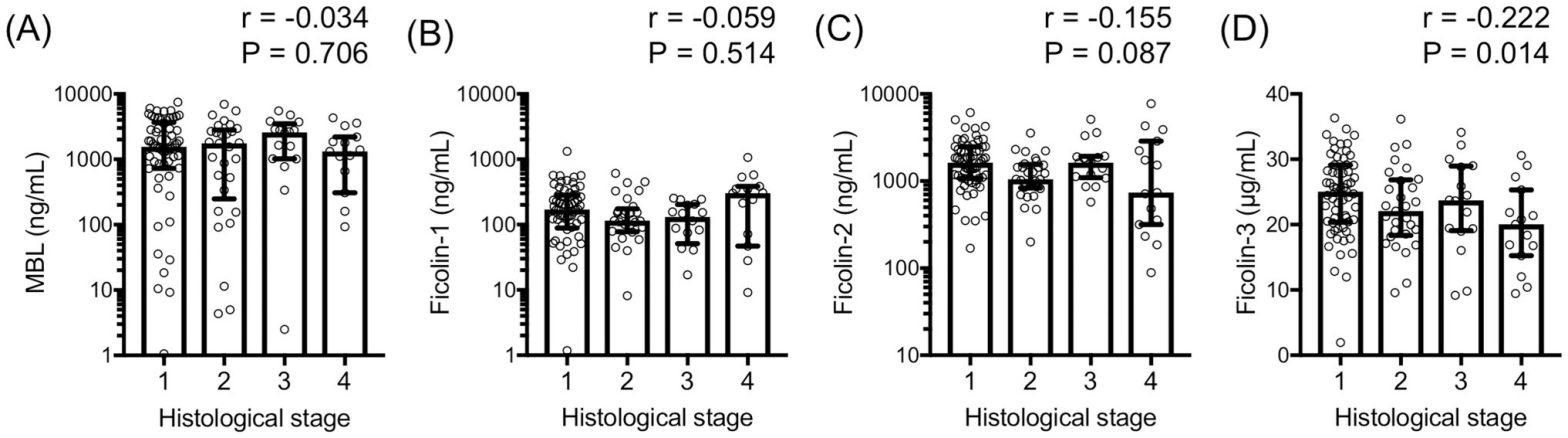
Serum levels of pattern recognition molecules according to histological stage showing (A) mannose-binding lectin (MBL), (B) ficolin-1, (C) ficolin-2 and (D) ficolin-3 levels.

We analyzed the association between the PRMs and liver histological stages graded according to Scheuer’s classification. Levels of ficolin-3 were negatively correlated with histological stage (stage 1, 25.0 μg/mL; stage 2, 22.0 μg/mL; stage 3, 23.7 μg/mL; and stage 4, 20.0 μg/mL. r = -0.222, P = 0.014). No significant correlation was observed between the histological stages and other PRM levels, such as MBL (stage 1, 1561 ng/mL; stage 2, 1755 ng/mL; stage 3, 2576 ng/mL; and stage 4, 1322 ng/mL. r = -0.034, P = 0.706), ficolin-1 (stage 1, 170 ng/mL; stage 2, 115 ng/mL; stage 3, 131 ng/mL; and stage 4, 303 ng/mL. r = -0.059, P = 0.514), and ficolin-2 (stage 1, 1624 ng/mL; stage 2, 1049 ng/mL; stage 3, 1628 ng/mL; and stage 4, 738 ng/mL. r = -0.155, P = 0.087) (Fig 4).

### Association between pattern recognition molecules and the development of cirrhosis-related conditions

**Fig 5:**
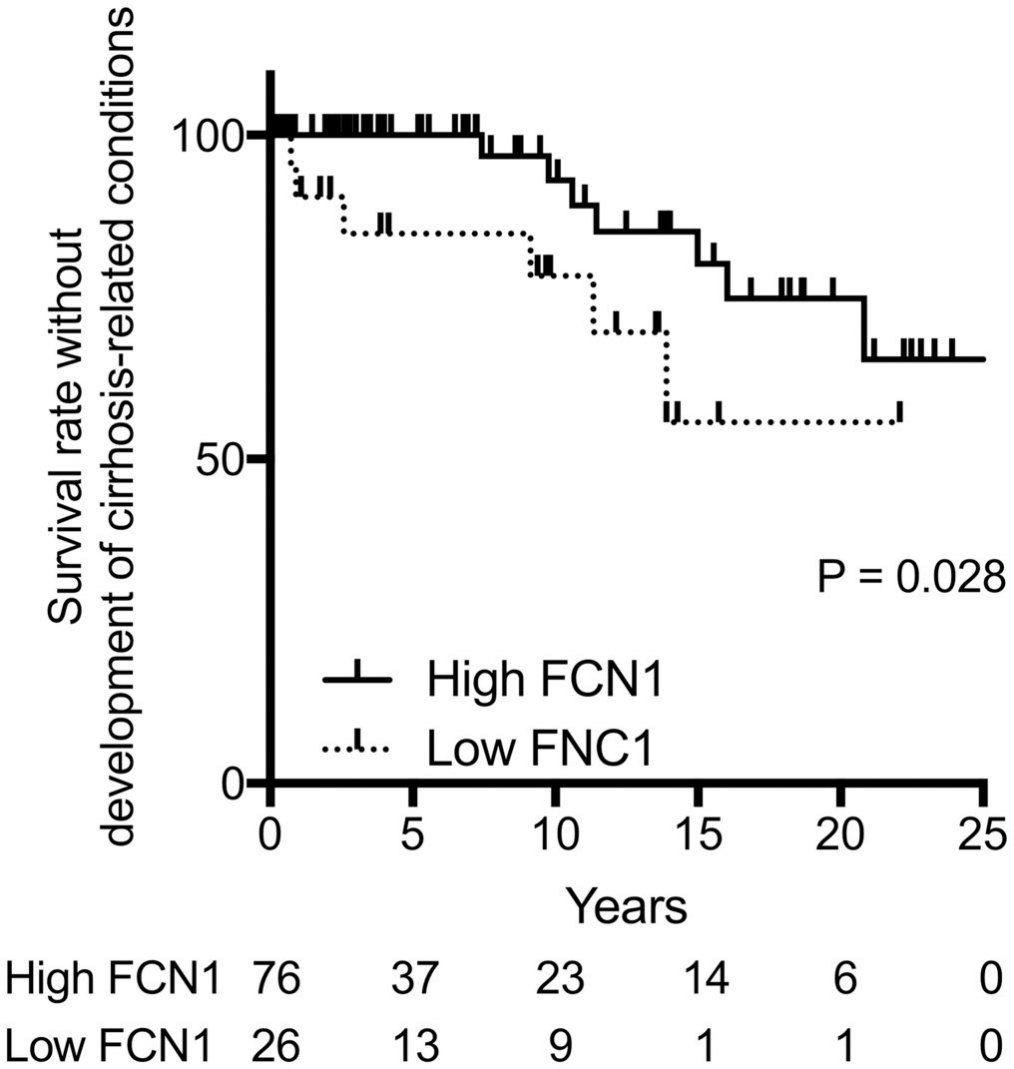
Kaplan-Meyer plots for the development of cirrhosis-related conditions. (A) The development of cirrhosis-related conditions was compared between the patients with high ficolin-1 levels and those with low ficolin-1 levels (The cut-off value of ficolin-1 is 77 ng/mL). FCN1, ficolin-1.

We assessed the association between the PRMs and the development of cirrhosis-related conditions using multivariate Cox proportional hazards models (Table 2). These models included histological stage according to Scheuer’s classification, ALP and each PRM level. The multivariate analyses showed that ficolin-1 levels were significantly associated with the development of cirrhosis-related conditions independent of histological stage and ALP levels (Hazard ratio, 0.933; 95% confidence interval, 0.875–0.994, P = 0.032). MBL, ficolin-2 and ficolin-3 did not show significant associations with the development of cirrhosis-related conditions. Kaplan-Meyer analysis of the development of cirrhosis-related conditions was performed using 25th percentile of ficolin-1 levels (cut-off value: 77 ng/mL). The Kaplan-Meyer plot showed a higher rate of development of cirrhosis-related conditions in the patients with low ficolin-1 levels than that in those with high ficolin-1 levels (log-rank; P = 0.028) (Fig 5).

**Table 2:** Association between pattern recognition molecules and the development of cirrhosis-related conditions.

	Hazard ratio	95% CI	P
MBL*, μg/mL	1.000	0.999–1.000	0.638
Ficolin-1*, 10ng/mL	0.933	0.875–0.994	0.032
Ficolin-2*, μg/mL	0.830	0.456–1.511	0.543
Ficolin-3*, μg/mL	0.978	0.908–1.054	0.562

MBL, mannose-binding lectin; CI, Confidence interval.

* Adjusted alkaline phosphatase and histological stage according to Scheuer staging system using multivariate Cox proportional hazards model.

## Discussion

The results of the present study demonstrate that serum levels of ficolin-1 was associated with the development of cirrhosis-related conditions in patients with PBC. The association between ficolin-1 and the development of cirrhosis-related conditions was independent of histological stage and ALP, which are predictors of prognosis in patients with PBC [20, 21]. All PRMs analyzed in the current study are known as initiators of lectin pathway activation in the complement system; however, only ficolin-1 showed associations with disease progression of PBC in the current study. Our results suggest that the contribution of ficolin-1 to the pathophysiology of PBC is different from those of the other three PRMs in the lectin pathway.

We measured levels of PRMs of lectin pathway, MBL, ficolin-1, ficolin-2, and ficolin-3. There have been several reports about the association between PRMs and disease activity of autoimmune diseases. High serum MBL level was reported to be associated with thrombocytopenia and seizure in patients with SLE [22], and serum MBL was reported to be positively correlated with Birmingham Vasculitis Activity Score in patients with antineutrophil cytoplasmic antibody-associated vasculitis [23]. In addition, serum ficolin-1 was positively correlated with disease activity in patients with rheumatoid arthritis (RA) [24] and juvenile idiopathic arthritis [25]. Upregulated mRNA levels of ficolin-1 in peripheral blood mononuclear cells, and a lot of ficolin-1–positive monocytes in the glomeruli in patients with microscopic polyangiitis have also been reported [26]. These findings suggest that complement PRMs are positively associated with inflammatory responses and disease activity in several autoimmune diseases. Further, high serum ficolin-1 was associated with poor prognosis in patients with systemic inflammatory response syndrome due to sepsis [27]. Excessive activation of the complement system can lead to harmful effects [28].

On the other hand, Hein et al. reported that serum ficolin-1 in patients with SLE was lower than that in healthy controls, and that serum ficolin-1 is negatively associated with disease activity in SLE patients [29]. Serum ficolin-2 in SLE patients has also been reported to be lower than that healthy controls, and low ficolin-2 levels have been associated with lupus nephritis or thrombocytopenia in patients with SLE [30, 31]. These findings suggest that a decrease of complement PRMs can also show disease activity. SLE patients have often been reported to show hypocomplementemia due to complement activation, and there has been deposition of complement at the site of injury site [32]. C1q, the initiator of classical complement pathway, shows the ability of the clearance of self-antigens by apoptotic cells [33]. C1q-deficient mice developed lupus live nephritis [34], and macrophage in C1q-deficient mice showed failure in apoptotic cells clearance [35]. Interestingly, Troldborg et al. reported that ficolin-3 deficiency was risk of SLE [36]. Moreover, in a study by Plawecki et al., anti-ficolin-3 antibodies were detected as positive in 37% of SLE patients, and the levels of anti-ficolin-3 antibodies were higher in patients with active than quiescent disease [37]. These reports suggest that insufficient levels of complement PRM in the lectin pathway can result in dysfunction of apoptotic cell clearance. It has been reported that apoptotic biliary epithelial cell preserved PDC-E2 epitope, and resulted in local autoimmune attack in PBC [12]. Moreover, clearance of apoptotic cholangiocytes via phagocytosis by macrophage was reported to lead to restoration of normal liver architecture in mice mode of biliary fibrosis [38]. In the current study, low ficolin-1 levels were associated with disease progression in patients with PBC. This result may indicate inadequate clearance of apoptotic biliary epithelial cells due to insufficient ficolin-1 levels.

Ficolin is a unique lectin with a fibrinogen-like domain that is responsible for carbohydrate recognition [39]. Although ficolin-1 is a non-serum type ficolin that is mainly expressed in peripheral monocytes [40], it also presents in serum, albeit at lower levels than those of ficolin-2 or ficolin-3 [7]. Mouse ficolin-B, a homologue of human ficolin-1, present in a complex with MBL-associated serine proteases (MASP)-2, and this complex showed C4-deposition activity in immobilized N-acetylglucosamine [41]. Pentraxin-3 (PTX-3) is a long pentraxin that is produced by hematopoietic and stromal cells in response to inflammation [42]. Ficolin-1 can interact with PTX3 via its fibrinogen-like domain. The complex formation between ficolin-1 and PTX3 recognized apoptotic or necrotic cells and removed these cells [43]. In addition, PTX-3 levels are positively correlated with a model for end-stage liver disease and Child-Pugh scores in cirrhotic patients [44]. We previously reported that serum cell death markers were high in patients with PBC [45]. Thus, ficolin-1 may be associated with dead cell clearance in PBC. Binding specifications differ in each ficolin. Interestingly, ficolin-1 binds to sialic acid, which is a part of molecular definition of self [46]. Ficoln-1 recognize sialic acid on the surface of monocytes, and ficoln-1 is tethered to the surface of monocytes [47]. Interestingly, MASP-2 is not detected on the surface of monocytes. In addition, ficolin-1 has been reported to have a much lower ability of complement activation compared with two other ficolins [48]. These results suggest that ficolin-1 on the surface of monocytes can play important roles for innate immunity without complement activation. This speculation is consistent with previous reports that there was little complement deposition around the bile duct in PBC patients [49].

Although the roles of monocytes in PBC are not fully understood, there have been some reports about the association between monocytes and PBC. Shimoda et al. reported that monocytes were detected around injured bile ducts in PBC patients [50], and Mao et al. reported that peripheral blood monocytes in patients with PBC produce more pro-inflammatory cytokines than that of healthy controls [51]. Chemokine receptor type 2 (CCR2) is a chemokine receptor which mediates migration of monocytes to the site of inflammation. Using a PBC mouse model, Reuveni et al showed that CCR2-deficient mice had mild cholangitis compared with wild-type mice [52]. Further, a dual CCR2/CCR5 antagonist (cenicriviroc) treatment improves fibrosis and inflammation in the liver in PBC mouse model. Serum macrophage activation markers, soluble CD163 and mannose receptor, are reported to be biomarkers of severity and prognosis in patients with PBC [53]. Taken together, the results of these reports suggest the important roles of macrophages and monocytes in patients with PBC. Ficolin-1 is mainly produced by macrophages and monocytes. Therefore, serum ficolin-1 may be associated with the roles of macrophages and monocytes in patients with PBC.

Interaction between ficolin-1 and T cells has been reported; the up-regulation of sialic acid expression has been observed on the surface of activated T cells, as has an increase in ficolin-1 binding to activated T cells [47], which play important roles in the pathophysiology of PBC [54]. T cell response is associated with injury of the biliary epithelial cells that line the intrahepatic bile ducts, resulting in inflammation and destruction of those ducts [55]. Ficolin-1 may be associated with inflammatory responses mediated by T cells.

In the current study, ficolin-1 was positively correlated with ALP, and low ficolin-1 was associated with poor prognosis independent of ALP levels in multivariate analysis. High ALP has been reported to be associated with poor prognosis in patients with PBC [21]. The serum activities of ALP after UDCA treatment is associated with disease progression [56]. Our results suggest the protective roles of ficolin-1 via dead cell clearance in PBC. On the other hand, high levels of ficolin-1 in patients with rheumatoid arthritis have been reported [10], and anti-ficolin-1 Ab ameliorated symptom of a mouse model of collagen antibody-induced arthritis [24]. It is known that the complement system plays multiple roles in homeostasis and disease, and acts as double-edged sword [6, 57]. Further studies are needed to confirm the association between ficolin-1 and the pathophysiology of PBC. Negative correlation between MBL and ALT may reflect consumption, as well as other disease [58]. Ficolin-2 was correlated with Alb, and ficolin-3 was negatively correlated with Alb and PT. These negative correlations suggest a decrease of production due to liver dysfunction, because ficolin-2 and ficolin-3 were mainly produced in the liver, as previously reported [7, 59].

Levels of ficolin-3 were significantly correlated with histological stage, albumin and prothrombin time in patients with PBC in the present study. Although there was no significant association, the ficolin-2 levels in PBC patients with stage 4 were low. Liver fibrosis is associated with a decrease in complement factors, because most complement factors are produced in the liver [7]. Ficolin-2 and ficolin-3 are also produced in the liver. Low levels of ficolin are predictors of development of bacterial infections in cirrhotic patients [59]. PBC patients with advanced histological stage may be associated with the development of infection in patients with PBC, as previously reported.

The current study has some limitations. First, we did not analyze the histological findings of PRM deposition in the liver. We should confirm the presence of ficolin-1 binding to liver tissue or lymphocytes in future studies. Second, we did not investigate the gene polymorphisms of PRMs. Levels of PRMs have been reported to be closely associated with gene polymorphisms [7]. Third, the present study was a retrospective single center analysis, with a small sample size. Future research should include analysis of a prospectively efficient sample size, with investigation of polymorphisms. Fourth, we did not assess the association between biochemical response to UDCA treatment and PRMs. There have been several criteria for response to treatment [2], which are useful for predicting prognosis in patients with PBC, and are important to consider when selecting the appropriate treatment.

In summary, low ficolin-1 levels were associated with PBC disease progression independent of histological stage and ALP levels, and ficolin-3 levels were negatively correlated with histological stage. These results suggest that there is an association between PRMs and PBC pathophysiology. Elucidation of the contribution of PRMs in the complement system to PBC would help in the understanding of PBC’s pathophysiology.
